# Interdisciplinary Strategies to Reduce Surgical Infectious Risk in the Operating Theater: Protocol for Scoping Review

**DOI:** 10.2196/67660

**Published:** 2025-02-12

**Authors:** Dominique Joubert, Sylvain Boloré, Carelle Baroni, Anne-Sophie Hans, Aline Wasser, Selin Kivrak, Audrey Murat-Ringot, Claude Dussart

**Affiliations:** 1 Health Care Directorate University Hospitals of Geneva Geneva Switzerland; 2 Laboratory “Health, Systemic, Process” (P2S), UR4129 University Claude Bernard Lyon 1 University of Lyon Lyon France; 3 Geneva School of Health Sciences HES-SO University of Applied Sciences and Arts Western Switzerland Geneva Switzerland; 4 Department of Ophthalmology University Hospitals of Geneva Geneva Switzerland; 5 Department of Anaesthesiology, Clinical Pharmacology, Intensive Care and Emergency Medicine Geneva University Hospitals Geneva Switzerland; 6 Institute of Higher Education and Research in Healthcare - IUFRS Lausanne University Hospital (CHUV) University of Lausanne (UNIL) Lausanne Switzerland; 7 Hospices Civils de Lyon Lyon France

**Keywords:** surgical site infection, infection prevention, interdisciplinary strategies, surgical team, operating room, standardized operating procedures

## Abstract

**Background:**

Surgical site infections (SSIs) represent one of the most prevalent and significant complications associated with surgical procedures, often leading to prolonged hospitalization and delayed patient recovery. While recent international consensus guidelines have proposed evidence-based strategies to mitigate SSIs, they fall short in addressing the efficient and interdisciplinary implementation of these measures within the operating theater. Consequently, further research is required to identify and evaluate optimal interdisciplinary organizational approaches for the prevention of SSIs.

**Objective:**

This study aims to map the scope, diversity, and nature of research on interdisciplinary strategies aimed at reducing SSIs and to analyze the impact of interdisciplinary on the effectiveness of preventive interventions.

**Methods:**

Using the Joanna Briggs Institute (JBI) methodology for scoping reviews, a comprehensive search will be conducted across databases including Embase (encompassing MEDLINE and PubMed-not-MEDLINE), CINAHL, and the Cochrane Library, supplemented by manual searches of reference lists from included papers. This review targets studies published between 2016 and 2024, aligning with the World Health Organization’s 2016 SSI prevention guidelines, which introduced significant advancements in practice and remain the global benchmark. Only studies published in English or French will be considered. Around 5 reviewers independently distributed the included papers for detailed reading and data extraction, while the lead author concurrently and independently reviewed all papers. Inclusion criteria follow the Participants, Concept, and Context (PCC) framework, specifying that the eligible population comprises surgical teams. The primary concept of interest is interdisciplinary strategies aimed at preventing infection risk. The context focuses on adult surgical procedures within the operating room during turnover periods. Studies using experimental, quasi-experimental, preexperimental, observational, case-control, or cross-sectional designs will be included.

**Results:**

From the 1679 papers initially identified, 45 were selected for detailed analysis by 5 reviewers, with the selection process completed by November 2024.

**Conclusions:**

Emerging interdisciplinary strategies demonstrate significant potential in reducing the incidence of SSIs. This initiative forms part of a broader global project focused on codeveloping standardized protocols for preoperative preparation within the operating room to mitigate SSI risks. The findings of this scoping review will serve as the foundation for a subsequent qualitative survey and a pre-post quasi-experimental quantitative study to evaluate the integration and effectiveness of these strategies in clinical practice. The review protocol will be formally registered in the Open Science Framework (OSF) in 2024.

**International Registered Report Identifier (IRRID):**

DERR1-10.2196/67660

## Introduction

### Background

The transition to advanced health care technologies is inherently linked to heightened patient safety risks within acute care hospital settings [1]. Operating theaters are environments where multiple risk factors converge, contributing to adverse or severe events. Among these, the risk of infection remains a primary concern and a persistent daily challenge, as infections represent a leading cause of preventable hospital mortality [2]. These infections contribute substantially to morbidity and mortality while also imposing significant additional burdens on health care costs [3]. In Switzerland, surgical site infections (SSIs) are the most prevalent type of health care–associated infection within medical facilities [4].

Adhering to evidence-based practice (EBP) has the potential to significantly reduce infection rates, with the corresponding levels of evidence for SSIs already well-documented in the literature [5]. In the operating theater, the implementation of both standard and procedure-specific precautions is crucial for effective infection prevention [6].

SSIs are nosocomial in nature, occurring after surgical procedures, and primarily influenced by pre-, intra-, and postoperative conditions, host immune response, and surgical cleanliness. Pathogen transmission predominantly occurs through contact, with health care workers’ hands serving as a major vector. Effective hand hygiene is paramount in preventing infections, particularly in acute care settings, where it plays a critical role in reducing the incidence of bacteremia, urinary tract infections, SSIs, and ventilator-associated pneumonia [3,7].

Key strategies include maintaining high standards in medical device disinfection, bio cleaning, and surface cleaning to minimize infection risks [8]. Monitoring adherence to disinfection protocols during turnover periods is essential for evaluating interdisciplinary coordination and minimizing contamination risks [9]. Adherence to current guidelines is crucial, given the rapidly evolving nature of EBP, as many practices once deemed acceptable are now considered contraindicated [10,11].

The World Health Organization (WHO) and Society for Healthcare Epidemiology of America (SHEA) recommendations for SSI prevention differ slightly in the level of evidence [2,10]. However, consensus exists regarding key practices, such as appropriate hair removal, surgical hand preparation, the use of alcohol-based skin antiseptics (eg, chlorhexidine gluconate solutions), optimal timing of antibiotic prophylaxis, and effective glycemic control [7,12,13]. Emphasis is also placed on the development of soft skills, the implementation of checklists, adherence to bundled care protocols, SSI monitoring, and providing feedback to operating theater staff members as key strategies for enhancing patient safety [5,12,14,15].

Low compliance with SSI prevention measures persists due to the challenges associated with implementing clinical guidelines effectively. Globally, only 29% of tertiary hospitals across 133 countries have established infection prevention and control (IPC) programs. In Switzerland, according to Swissmedic (2022), 93% of 35 surveyed institutions out of 300 reported deficiencies in critical areas, including procedural adherence, staff training, the presence of hygiene specialists, and cognitive dissonance within operating theater teams.

Operating theaters demand the coordinated efforts of surgeons, anesthetists, technicians, and nurses to deliver high-quality care. Effective IPC depends on robust collaboration, clear communication, cohesive teamwork, and streamlined logistics across various hospital disciplines [16]. The complex and highly technical environment of operating theaters, combined with the diverse professional backgrounds of team members, necessitates effective interprofessional collaboration, particularly in time-sensitive situations [17]. Managing human error in the perioperative period remains a major challenge [18-24].

Interprofessionality emphasizes practical collaboration among skilled professionals, while interdisciplinary focuses on integrating knowledge from diverse academic disciplines to address complex challenges. Both approaches are essential for effective IPC in health care settings.

Although expert guidelines from organizations such as WHO, SHEA, Infectious Diseases Society of America (IDSA), Association for Professionals in Infection Control and Epidemiology (APIC), and American Hospital Association (AHA) provide a strong foundation, they lack sufficient specificity regarding the interdisciplinary applications required for SSI prevention in operating theaters [10]. A preliminary search revealed a lack of recent systematic reviews, underscoring the necessity of investigating and evaluating effective interdisciplinary strategies for reducing SSIs.

Review questions:

1. What are the interdisciplinary strategies to reduce SSIs in the operating theater?

2. What are its characteristics?

3. What improvements have been observed concerning the interventions and their evaluation?

4. Would it be possible to apply a model considering the culture and the local specificities?

### Study Aim

This scoping review seeks to map the scope, diversity, and nature of existing research on interdisciplinary strategies for reducing SSIs. The objective is to identify patterns, gaps, and innovations in the literature while evaluating how the integration of diverse disciplinary approaches enhances outcomes in SSI prevention.

## Methods

### Overview

The proposed scoping review will be conducted according to the Joanna Briggs Institute (JBI) methodology for scoping reviews [25] and the PRISMA-ScR (Preferred Reporting Items for Systematic Reviews and Meta-Analyses extension for Scoping Reviews) [26].

### Search Strategy

A 3-step search strategy will be used for this review. Initially, a preliminary limited search was conducted in MEDLINE (via PubMed) to identify relevant papers on the topic. The text words in the titles and abstracts of these papers, along with the index terms used to describe them, were extracted and cataloged for use as search terms. The second step involves a comprehensive search incorporating all identified keywords and index terms, tailored for each database and information source. Databases to be searched include CINAHL (Multimedia Appendix 1), Embase (Multimedia Appendix 2), MEDLINE (Multimedia Appendix 3), and the Cochrane Library (Multimedia Appendix 4).

The third step comprises a manual search of the reference lists of all included papers to identify additional studies meeting the inclusion criteria. This review focuses on studies published between 2016 and 2024, a period defined by the WHO’s designation of SSIs as a global priority and the release of its prevention guidelines. This time frame reflects significant advancements with outdated practices being phased out. Priority was given to studies adhering to these guidelines as no major updates have been introduced since their publication.

Systematic reviews conducted by organizations such as the Centers for Disease Control and Prevention, SHEA, and others broadly align with WHO recommendations, with variations primarily in evidence grading. Additional studies were identified through reference list searches of included papers. Only papers published in English or French were included in the review.

### Inclusion Criteria

The Participants, Concept, and Context (PCC) framework was used to guide the identification and selection of studies for inclusion in this review [25].

#### Participants

The eligible population comprises surgical teams, encompassing all professionals involved in patient preparation within the operating room immediately before surgery. This includes surgeons, anesthetists, anesthetic nurses, registered nurses, scrub nurses, instrument technicians, and nursing assistants. Papers will be included if they address the involvement of at least 3 distinct disciplines [6].

#### Concept

This review focuses on interdisciplinary strategies for IPC. Interdisciplinary in this context does not merely refer to the diverse characteristics of personnel, such as education or professional roles, but rather to a coordinated framework for joint action, exemplified by standard operating procedures. These interdisciplinary approaches leverage shared mental models and visual triggers to activate synchronization among team members. Such frameworks have been described and validated in other industries, such as task-sharing standard operating procedures used by Airbus in cockpit operations. Papers will be included if they report on at least one interdisciplinary strategy.

#### Context

The specific context is adult surgical procedures within operating theaters during turnover, defined as the period between the closure of one patient’s surgical wound and the incision of the next. This turnover period is critical for infection prevention through measures such as antibiotic prophylaxis and skin preparation. In adult surgeries, turnover often involves high-risk procedures, necessitating robust infection control strategies. Only studies that report interventions during turnover in adult surgical settings will be included; those focusing on pediatric surgery or unrelated contexts will be excluded. A significant body of EBP emphasizes reducing infection risks during this critical transitional phase.

### Types of Sources

This scoping review will include experimental and quasi-experimental study designs, such as randomized controlled trials, nonrandomized controlled trials, before-and-after studies, and interrupted time-series studies. In addition, analytical observational studies, including prospective and retrospective cohort studies, case-control studies, and analytical cross-sectional studies, will be considered for inclusion. Publications such as editorials, commentaries, letters, conference proceedings, and gray literature will be excluded as the focus is on identifying effective and validated interdisciplinary models.

### Study or Source of Evidence Selection

Following the search, all identified citations will be compiled and uploaded into EndNote 20 (Clarivate), where duplicate entries will be removed. After conducting a pilot test, titles and abstracts will be screened by 2 independent reviewers to determine their alignment with the inclusion criteria. Study selection will involve a dual-review process of titles and abstracts, followed by a thorough analysis of the full text. In cases of discrepancies, a third reviewer will be consulted to reach a consensus. Full-text versions of potentially relevant sources will be retrieved and their citation details will be imported into the JBI System for the Unified Management, Assessment, and Review of Information (JBI) [27]. Around 5 reviewers will independently evaluate the full text of selected citations against the inclusion criteria. Reasons for excluding sources that do not meet these criteria will be systematically documented and reported in the scoping review. To ensure thoroughness and consistency, all reviewers will collectively read and analyze a subset of the included papers, while the lead author will independently review all included papers to guarantee a comprehensive and unbiased assessment.

Any disagreements among reviewers at any stage of the selection process will be resolved through discussion or, if necessary, with the involvement of additional reviewers. The search results and study inclusion process will be fully documented in the final scoping review and presented using a PRISMA-ScR flow diagram.

### Data Extraction

A total of 5 reviewers divided the included papers among themselves for in-depth reading and data extraction, while the lead author independently reviewed all papers in parallel. In cases of confusion or disagreement, the extraction files were compared and discussed with the relevant reviewer to ensure accuracy and consensus. All reviewers used a custom-designed data extraction tool (Data extraction instrument, Multimedia Appendices 5-8; Textbox S1 in Multimedia Appendix 9) to systematically collect data from the studies included in the scoping review.

The data extracted includes detailed information on participants, the concept under investigation, the context, study methodologies, and key findings pertinent to the review question. In addition, general information about each study, as well as its methods, characteristics, and results, will be documented to provide a comprehensive overview. The draft data extraction tool will be adapted and refined as necessary to ensure the accurate extraction of data from each included evidence source. Any modifications made to the tool will be documented in detail within the scoping review. Disagreements among reviewers regarding data extraction will be resolved through discussion or, if required, with the involvement of an additional reviewer. Where appropriate, the authors of the included papers will be contacted to obtain missing or supplementary data.

## Results

From the 1679 papers initially identified, 45 were selected for detailed analysis by 5 reviewers, with the selection process completed by November 2024.

The interprofessional perspectives and interdisciplinary interventional components of various strategies aimed at reducing SSIs in the operating theater will be thoroughly detailed. The review will specify whether these interventions were introduced and developed within the framework of a particular implementation model or care concept. In addition, key elements of the triggers synchronization bundle, which facilitate safe organizational practices, proper procedural sequencing, and the delivery of high-quality care, will be highlighted. At the beginning of the year 2025, the identified preventive strategies will be presented through narrative syntheses and summary tables, supplemented by graphs illustrating the distribution of studies and emerging trends related to the scoping review questions. General extraction information will be summarized in a descriptive table (Textbox S1 in Multimedia Appendix 9) and further detailed (Multimedia Appendix 5) for each category. These categories include years of publication, study locations, study designs, sample sizes, and participant demographics. In addition, for the observed interventions, it will be specified whether they fully or partially align with EBP and international recommendations.

## Discussion

### Overview

We are conducting a comprehensive review of the literature to identify the most effective multidisciplinary models implemented in operating theaters for reducing SSIs. Our objectives are to characterize the key features of these models, evaluate their impact on organizational outcomes, and explore their association with SSI rates. While it is likely that we will identify efficient models, establishing a definitive hierarchy of their effectiveness may be challenging due to the unique characteristics inherent to each surgical specialty. However, several interdisciplinary practices are expected to exert significant influence—either positively or negatively—on the incidence of SSIs.

Our goal is to propose a generalizable model applicable across the majority of surgical specialties, focusing on improving practices and enhancing communication within the operating room to reduce variability. Furthermore, we aim to identify a replicable model suitable for implementation in Switzerland, emphasizing the importance of a collaborative approach adapted to the specific constraints of health care settings.

Evidence-based interdisciplinary strategies have demonstrated effectiveness in reducing SSIs. Research indicates that the successful implementation of care bundles is frequently associated with improvements in process outcomes rather than direct patient outcomes. Nonetheless, patient outcomes consistently show significant reductions in postoperative infection rates when interdisciplinary interventions are rigorously applied [28]. A significant challenge lies in ensuring the consistent engagement of health care professionals, particularly surgeons, in implementing interdisciplinary strategies. The studies reviewed predominantly offer moderate-quality evidence, highlighting the pressing need for standardized approaches and tools to enhance the effectiveness and reproducibility of interventions [29]. However, the concept of interdisciplinary remains underexplored in the existing literature. While support and training programs are frequently emphasized, no singular pivotal factor emerges as decisively shaping the health care system or fostering interdisciplinary collaboration. Notably, critical principles such as decompartmentalization and care synchronization—core components of the cognitive model bundle developed by the Airbus industry to optimize team efficiency—have yet to be systematically identified or applied within this context.

### Limitations

This scoping review is restricted to adult patients and the included papers are limited to those published in English and French, which may result in the exclusion of some relevant evidence. In addition, in several selected studies, the outcome measures are not reported, complicating the evaluation of the interventions’ impact on SSI rates. Even when SSI rates are provided, the variability in monitoring methods must be taken into account, as it can influence the interpretation and comparability of results.

### Conclusions

Emerging interdisciplinary strategies demonstrate promising potential for the prevention of SSIs and could be effectively replicated in Switzerland through a tailored implementation model. The bundles of care analyzed in this review appear to be robust in structure; however, the quality of interdisciplinary and their implementation in the operating theater remains challenging to evaluate, with adherence to recommended practices consistently falling short of optimal levels. The need for standardization of these bundles is frequently emphasized across studies.

Notably, this review did not reveal significant innovations, underscoring a gap in the literature. It forms part of a global initiative to cocreate a standardized approach to preoperative preparation in the operating room to reduce SSIs. While studies often highlight what should be done, they rarely address how to implement these measures effectively, pointing to an urgent need for innovative and actionable strategies. This presents an opportunity to develop a coconstructed model in collaboration with surgical teams, ensuring its feasibility and effectiveness through evaluation in real-world clinical settings.

## Appendix

Multimedia Appendix 1 CINAHL Search strategy from Dec 2016 to May 2024.

Multimedia Appendix 2 Embase Search strategy from Dec 2016 to May 2024.

Multimedia Appendix 3 MEDLINE Search strategy from Dec 2016 to May 2024.

Multimedia Appendix 4 Cochrane Library Search strategy from Dec 2016 to May 2024.

Multimedia Appendix 5 Data extraction instrument.

Multimedia Appendix 6 Data extraction instrument Characteristics 1.

Multimedia Appendix 7 Data extraction instrument Characteristics 2.

Multimedia Appendix 8 Data extraction instrument Characteristics 3.

Multimedia Appendix 9 Data extracted from studies.

## Abbreviations

AHA: American Hospital Association
APIC: Association for Professionals in Infection Control and Epidemiology
EBP: evidence-based practice
IDSA: Infectious Diseases Society of America
IPC: infection prevention and control
JBI: Joanna Briggs Institute
OSF: Open Science Framework
PCC: Participants, Concept, and Context
PRISMA-ScR: Preferred Reporting Items for Systematic Reviews and Meta-Analyses extension for Scoping Reviews
SHEA: Society for Healthcare Epidemiology of America
SSI: surgical site infection
WHO: World Health Organization

## References

[1] Rosalind Ievins Rosemary Sudan, Rebecca Bierman (2015). Guide pédagogique pour la sécurité des patients: édition multiprofessionnelle. Organisation mondiale de la Santé.

[2] Calderwood MS, Anderson DJ, Bratzler DW, Dellinger EP, Garcia-Houchins S, Maragakis LL, Nyquist AC, Perkins KM, Preas MA, Saiman L, Schaffzin JK, Schweizer M, Yokoe DS, Kaye KS (2023). Strategies to prevent surgical site infections in acute-care hospitals: 2022 update. Infect Control Hosp Epidemiol.

[3] Moszkowicz D, Hobeika C, Collard M, Bruzzi M, Beghdadi N, Catry J, Duchalais E, Manceau G, Voron T, Lakkis Z, Allard AM, Cauchy F, Maggiori L (2019). Operating room hygiene: clinical practice guidelines SFCD-ACHBT. J Visc Surg.

[4] Zingg PWD, Metsini A, Gardiol C, Balmelli C, Behnke M, Troillet N, Widmer A, Pittet D (2019). Second national point prevalence survey of healthcare-associated infections and antimicrobial use in Swiss acute care hospitals. Euro Surveill.

[5] Kuster SP, Eisenring MC, Sax H, Troillet N, Swissnoso (2017). Structure, process, and outcome quality of surgical site infection surveillance in Switzerland. Infect Control Hosp Epidemiol.

[6] Allegranzi B, Bischoff P, de Jonge S, Kubilay NZ, Zayed B, Gomes SM, Abbas M, Atema JJ, Gans S, van Rijen M, Boermeester MA, Egger M, Kluytmans J, Pittet D, Solomkin JS, WHO Guidelines Development Group (2016). New WHO recommendations on preoperative measures for surgical site infection prevention: an evidence-based global perspective. Lancet Infect Dis.

[7] (Department of Service Delivery and Safety, WHO (2018). Global Guidelines for the Prevention of Surgical Site Infection. World Health Organization.

[8] Munoz-Price LS, Birnbach DJ, Lubarsky DA, Arheart KL, Fajardo-Aquino Y, Rosalsky M, Cleary T, Depascale D, Coro G, Namias N, Carling P (2012). Decreasing operating room environmental pathogen contamination through improved cleaning practice. Infect Control Hosp Epidemiol.

[9] Yezli S, Barbut F, Otter JA (2014). Surface contamination in operating rooms: a risk for transmission of pathogens?. Surg Infect (Larchmt).

[10] Leaper DJ, Edmiston CE (2017). World Health Organization: global guidelines for the prevention of surgical site infection. J Hosp Infect.

[11] Solomkin JS, Mazuski J, Blanchard JC, Itani KM, Ricks P, Dellinger EP, Allen G, Kelz R, Reinke CE, Berríos-Torres SI (2017). Introduction to the centers for disease control and prevention and the healthcare infection control practices advisory committee guideline for the prevention of surgical site infections. Surg Infect (Larchmt).

[12] Lefebvre A, Saliou P, Lucet JC, Mimoz O, Keita-Perse O, Grandbastien B, Bruyère F, Boisrenoult P, Lepelletier D, Aho-Glélé L S, French Study Group for the Preoperative Prevention of Surgical Site Infections (2015). Preoperative hair removal and surgical site infections: network meta-analysis of randomized controlled trials. J Hosp Infect.

[13] Swissnoso (2018). Swissnoso Cndpdi. Standard guidelines: Preoperative skin disinfection. Swissnoso; 2018. Swissnoso Cndpdi. Standard guidelines: Preoperative skin disinfection. Swissnoso; 2018.

[14] Link T (2022). Guidelines in practice: preoperative patient skin antisepsis. AORN J.

[15] Harders M, Malangoni MA, Weight S, Sidhu T (2006). Improving operating room efficiency through process redesign. Surgery.

[16] Avery 3rd DM, Matullo KS (2014). The efficiency of a dedicated staff on operating room turnover time in hand surgery. J Hand Surg Am.

[17] Cullati S, Le Du S, Raë AC, Micallef M, Khabiri E, Ourahmoune A, Boireaux A, Licker M, Chopard P (2013). Is the surgical safety checklist successfully conducted? An observational study of social interactions in the operating rooms of a tertiary hospital. BMJ Qual Saf.

[18] Wilson A (2019). Creating and applying shared mental models in the operating room. J Perioper Nurs.

[19] Garosi E, Kalantari R, Zanjirani Farahani A, Zuaktafi M, Hosseinzadeh Roknabadi E, Bakhshi E (2020). Concerns about verbal communication in the operating room: a field study. Hum Factors.

[20] Lingard L, Espin S, Whyte S, Regehr G, Baker GR, Reznick R, Bohnen J, Orser B, Doran D, Grober E (2004). Communication failures in the operating room: an observational classification of recurrent types and effects. Qual Saf Health Care.

[21] Thiels CA, Lal TM, Nienow JM, Pasupathy KS, Blocker RC, Aho JM, Morgenthaler TI, Cima RR, Hallbeck S, Bingener J (2015). Surgical never events and contributing human factors. Surgery.

[22] Wheelock A, Suliman A, Wharton R, Babu ED, Hull L, Vincent C, Sevdalis N, Arora S (2015). The impact of operating room distractions on stress, workload, and teamwork. Ann Surg.

[23] Sockeel P, Chatelain E, Massoure MP, David P, Chapellier X, Buffat S (2009). Surgeons can learn from pilots: human factors in surgery. J Chir (Paris).

[24] Kohn LT, Corrigan JM, Donaldson MS, Institute of Medicine (US) Committee on Quality of Health Care in America (2000). To Err Is Human: Building a Safer Health System.

[25] Peters MDJ, Marnie C, Tricco AC, Pollock D, Munn Z, Alexander L, McInerney P, Godfrey CM, Khalil H (2020). Updated methodological guidance for the conduct of scoping reviews. JBI Evid Synth.

[26] Tricco AC, Lillie E, Zarin W, O'Brien KK, Colquhoun H, Levac D, Moher D, Peters MDJ, Horsley T, Weeks L, Hempel S, Akl EA, Chang C, McGowan J, Stewart L, Hartling L, Aldcroft A, Wilson MG, Garritty C, Lewin S, Godfrey CM, Macdonald MT, Langlois EV, Soares-Weiser K, Moriarty J, Clifford T, Tunçalp Ö, Straus SE (2018). PRISMA extension for Scoping Reviews (PRISMA-ScR): checklist and explanation. Ann Intern Med.

[27] Munn Z, Aromataris E, Tufanaru C, Stern C, Porritt K, Farrow J, Lockwood C, Stephenson M, Moola S, Lizarondo L, McArthur A, Peters M, Pearson A, Jordan Z (2019). The development of software to support multiple systematic review types: the joanna briggs institute system for the unified management, assessment and review of information (JBI SUMARI). Int J Evid Based Healthc.

[28] Camperlengo L, Spencer M, Graves P, Danker W, Edmiston CE (2023). Effectiveness versus uptake: the challenges of implementing evidence-based strategies to reduce surgical site infection in patients with colon surgeries. Surg Infect (Larchmt).

[29] Arnal-Velasco D, Paz-Martín D (2022). Extension of patient safety initiatives to perioperative care. Curr Opin Anaesthesiol.

