# Supplementary material for: Interdisciplinary Strategies to Reduce Surgical Infectious Risk in the Operating Theater: Protocol for Scoping Review
Source: JMIR Res Protoc. 2025 Feb 12;14:e67660. doi: 10.2196/67660 (PMC11888008; doi:10.2196/67660)
Supplement: Multimedia Appendix 1 [file resprot_v14i1e67660_app1.docx]

## Multimedia Appendix 1: CINAHL Search strategy from Dec 2016 to May 2024

*CINAHL: 10.06.2024*

| Concept | # | Research strategy | Number of  references identified |
| --- | --- | --- | --- |
| A | 1 | (MH "Operating Room Personnel+") OR (MH "Operating Room Nurses") OR (MH "Surgical Technologists") OR (MH "Anesthesia Nursing") OR (MH "Scrub Nurses") OR (MH "Circulating Nurses") | 10144 |
| A | 2 | ("Surgical Team*" OR "Surgeon*" OR "Anaesthetist*" OR "Anaesthetic Nurse*" OR " Instrument Technician*" OR "scrub Nurse*" OR "Nursing Assistant*" OR “circulating nurse*”) | 41479 |
| A | 3 | 1 OR 2 | 44079 |
|  | 4 | (MH "Surgical Wound Infection") OR (MH "Infection/PC") OR (MH "Sepsis/PC") | 7461 |
| B | 5 | (“Infection Prevention” OR “Infectious Risk” OR “healthcare associated infection*” OR “surgical site infection*” OR “SSI” OR “infection control” OR “Infectious risk management” OR “strategies to prevent health care associated infection”) | 24769 |
| B | 6 | 4 OR 5 | 27037 |
| B | 7 | (MH "Multidisciplinary Care Team") | 20963 |
| B | 8 | (“Interdisciplinary Strateg* “ OR “Interdisciplinary practice*" OR "Interprofessional” OR “Multidisciplinary” OR “Multiprofessional” OR ”Workflow” OR “bundle” OR “bundle to prevent SSI” OR “Bundle of care“ OR “Standard operating procedures” OR tasksharing” OR “interdisciplinary strategy”) | 68167 |
| B | 9 | 7 OR 8 | 68167 |
| A+B | 10 | 3 AND 6 AND 9 | 98 |
