# Supplementary material for: Interdisciplinary Strategies to Reduce Surgical Infectious Risk in the Operating Theater: Protocol for Scoping Review
Source: JMIR Res Protoc. 2025 Feb 12;14:e67660. doi: 10.2196/67660 (PMC11888008; doi:10.2196/67660)
Supplement: Multimedia Appendix 2 [file resprot_v14i1e67660_app2.docx]

## Multimedia Appendix 2 Embase Search strategy from Dec 2016 to May 2024

*Embase: 11.06.2024*

| Concept | # | Research strategy | Number of  references identified |
| --- | --- | --- | --- |
| A | 1 | ('operating room technicians'/exp OR 'operating room nursing'/exp OR 'surgeons'/exp) | 16462 |
| A | 2 | ('Surgical Team*' OR Surgeon* OR Anaesthetist* OR 'Anaesthetic Nurse*' OR ' Instrument Technician*' OR 'scrub Nurse*' OR 'Nursing Assistant*' OR 'circulating nurse*') | 337962 |
| A | 3 | 1 OR 2 | 344299 |
|  | 4 | ('Surgical Wound Infection'/exp OR sepsis/exp OR 'infection control'/exp) | 233760 |
| B | 5 | ('infection prevention' OR 'infectious risk' OR 'healthcare associated infection*' OR 'surgical site infection*' OR ssi OR 'infection control' OR 'infectious risk management') | 119388 |
| B | 6 | 4 OR 5 | 299328 |
| B | 7 | ('Interdisciplinary Communication'/exp) | 4235 |
| B | 8 | ('interdisciplinary strateg*' OR 'interdisciplinary practice*' OR interprofessional OR multidisciplinary OR multiprofessional OR workflow OR bundle OR 'bundle to prevent SSI' OR 'bundle of care' OR 'standard operating procedures' OR tasksharing OR (interdisciplinary AND (studies OR study)) OR (interdisciplinary AND (strategie OR strategies OR strategy OR 'strategy s'))) | 440976 |
| B | 9 | 7 OR 8 | 442582 |
| A+B | 10 | 3 AND 6 AND 9 | 1022 |
