# Supplementary material for: Interdisciplinary Strategies to Reduce Surgical Infectious Risk in the Operating Theater: Protocol for Scoping Review
Source: JMIR Res Protoc. 2025 Feb 12;14:e67660. doi: 10.2196/67660 (PMC11888008; doi:10.2196/67660)
Supplement: Multimedia Appendix 3 [file resprot_v14i1e67660_app3.docx]

## Multimedia Appendix 3 MEDLINE Search strategy from Dec 2016 to May 2024

*MEDLINE 11.06.2024*

|  | 1 | ("Operating Room Technicians"[Mesh] OR "Operating Room Nursing"[Mesh] OR "Surgeons"[Mesh]) | 15924 |
| --- | --- | --- | --- |
| A | 2 | ("Surgical Team*" OR "Surgeon*" OR "Anaesthetist*" OR "Anaesthetic Nurse*" OR "Instrument Technician*" OR "scrub Nurse*" OR "Nursing Assistant*" OR “circulating nurse*”) | 160164 |
| A | 3 | 1 OR 2 | 161165 |
|  | 4 | ("Surgical Wound Infection"[Mesh] OR "sepsis"[MeSH] OR "Infection Control"[Mesh]) | 70437 |
| B | 5 | (“Infection Prevention” OR “Infectious Risk” OR “healthcare associated infection*” OR “surgical site infection*” OR “SSI” OR “infection control” OR “Infectious risk management”) | 62243 |
| B | 6 | 4 OR 5 | 114087 |
| B | 7 | ("Interdisciplinary Communication"[Mesh]) | 4781 |
| B | 8 | ('interdisciplinary strateg*' OR 'interdisciplinary practice*' OR interprofessional OR multidisciplinary OR multiprofessional OR workflow OR bundle OR 'bundle to prevent SSI' OR 'bundle of care' OR 'standard operating procedures' OR tasksharing OR (interdisciplinary AND (studies OR study)) OR (interdisciplinary AND (strategie OR strategies OR strategy OR 'strategy s')))) | 430184 |
| B | 9 | 7 OR 8 | 431498 |
| A+B | 10 | 3 AND 6 AND 9 | 873 |
