# Supplementary material for: Interdisciplinary Strategies to Reduce Surgical Infectious Risk in the Operating Theater: Protocol for Scoping Review
Source: JMIR Res Protoc. 2025 Feb 12;14:e67660. doi: 10.2196/67660 (PMC11888008; doi:10.2196/67660)
Supplement: Multimedia Appendix 4 [file resprot_v14i1e67660_app4.docx]

## Multimedia Appendix 4 Cochrane Library Search strategy from Dec 2016 to May 2024

*Cochrane Library 03.06.2024*

|  | 1 | ("Operating Room Technicians" OR "Operating Room Nursing" OR "Surgeons") | 436 |
| --- | --- | --- | --- |
| A | 2 | (("Surgical" NEXT Team*) OR Surgeon* OR Anaesthetist* OR ("Anaesthetic" NEXT Nurse*) OR ("Instrument" NEXT Technician*) OR ("scrub" NEXT Nurse*) OR ("Nursing" NEXT Assistant*) OR ("circulating" NEXT nurse*)) | 641 |
| A | 3 | 1 OR 2 | 643 |
|  | 4 | [mh "Surgical Wound Infection"] OR [mh infections] OR [mh sepsis] | 544 |
| B | 5 | ("Infection Prevention" OR "Infectious Risk" OR ("healthcare associated" NEXT infection*) OR ("surgical site" NEXT infection*) OR SSI OR "infection control" OR "Infectious risk management") | 210 |
| B | 6 | 4 OR 5 | 638 |
| B | 7 | ([mh "Interdisciplinary Communication"]) | 165 |
| B | 8 | (Interdisciplinary NEXT Strategy OR Interdisciplinary NEXT practice OR Interprofessional OR Multidisciplinary OR Multiprofessional OR Workflow OR bundle OR bundle NEXT SSI OR Bundle NEXT care OR Standard NEXT operating OR task sharing OR interdisciplinary NEXT strategy OR interdisciplinary NEXT strategies OR interdisciplinary NEXT study OR interdisciplinary NEXT studies) | 557 |
| B | 9 | 7 OR 8 | 557 |
| A+B | 10 | 3 AND 6 AND 9 | 27 |
