# Supplementary material for: Interdisciplinary Strategies to Reduce Surgical Infectious Risk in the Operating Theater: Protocol for Scoping Review
Source: JMIR Res Protoc. 2025 Feb 12;14:e67660. doi: 10.2196/67660 (PMC11888008; doi:10.2196/67660)
Supplement: Multimedia Appendix 5 [file resprot_v14i1e67660_app5.docx]

## Multimedia Appendix 5 Data extraction instrument

| **General information** | | | **Methods** | | |
| --- | --- | --- | --- | --- | --- |
| **Author’s name** | **Publication year** | **Country** | **Design** | **Participants** | **characteristic / sampling and analyse** |
|  |  |  | Randomized controlled trial | Surgeon | sizes/setting |
|  |  |  | Non-randomized controlled trial | Anaesthetist | Single Centre |
|  |  |  | Before and after | Anaesthetic Nurse | Multicentre |
|  |  |  |  | Instrument Technician | Interrupted time-series |
|  |  |  |  | Scrub Nurse |  |
|  |  |  |  | Nursing Assistant |  |
|  |  |  |  | Circulating nurse |  |
|  |  |  |  | Surgical Team |  |
