# Supplementary material for: Interdisciplinary Strategies to Reduce Surgical Infectious Risk in the Operating Theater: Protocol for Scoping Review
Source: JMIR Res Protoc. 2025 Feb 12;14:e67660. doi: 10.2196/67660 (PMC11888008; doi:10.2196/67660)
Supplement: Multimedia Appendix 6 [file resprot_v14i1e67660_app6.docx]

## Multimedia Appendix 6 Data extraction instrument Characteristics 1

| Characteristics 1 |  |  |  |
| --- | --- | --- | --- |
| **Location** | **Time around surgery** | **Team Framework/model** | **Patient partnership** |
| Operating room | Turnover | CRM | Patient inputs |
| Patient zone | intraoperative period | Team STEPPS | Exchange of information and co-operation with the patient prior to the induction of anaesthesia |
| Anaesthetic area | post operative period | Aeronautical SOPs+ |  |
| Turnover |  | Bundle of care |  |
| Post-surgery time |  | Checklist |  |
|  |  | Other industry model |  |
