# Supplementary material for: Interdisciplinary Strategies to Reduce Surgical Infectious Risk in the Operating Theater: Protocol for Scoping Review
Source: JMIR Res Protoc. 2025 Feb 12;14:e67660. doi: 10.2196/67660 (PMC11888008; doi:10.2196/67660)
Supplement: Multimedia Appendix 7 [file resprot_v14i1e67660_app7.docx]

## Multimedia Appendix 7 Data extraction instrument Characteristics 2

| **Characteristics 2** |  |  |  |
| --- | --- | --- | --- |
| **Implementation strategies** | **Triggers** | **Cognitive aid** | **Nursing Care concept** |
| Education and training | Synchronized care practice | Icon Graph | Strengths-based nursing (SBN) |
| Simulation training | Announcement | Pocket card | Patient satisfaction |
| E-Learning | Sonor or visual alarm | Pictogram | Multidisciplinary team (MDT) |
| Team coaching |  |  |  |
| Re-aim, CFIR, TDF Framework, EPIS |  |  |  |
