# Supplementary material for: Interdisciplinary Strategies to Reduce Surgical Infectious Risk in the Operating Theater: Protocol for Scoping Review
Source: JMIR Res Protoc. 2025 Feb 12;14:e67660. doi: 10.2196/67660 (PMC11888008; doi:10.2196/67660)
Supplement: Multimedia Appendix 8 [file resprot_v14i1e67660_app8.docx]

**Multimedia Appendix 8 Data extraction instrument Characteristics 3**

| **Characteristics 3** |  |  |  |
| --- | --- | --- | --- |
| **Outcome process** | **Outcome patient** | **Key finding** | **Results** |
| Adaptation of the model | SSI rate | Model Efficiency | Positive |
| Compliance with recommendation | Patient satisfaction | Innovation | negative |
| Adopting the care model | Patient morbitiy/mortality | Quality improvement | Level of proof |
|  | Length of stay | Skills and knowledge improvement |  |
|  | Number of days lost | Reproducibility in Switzerland |  |
