# Supplementary material for: Interdisciplinary Strategies to Reduce Surgical Infectious Risk in the Operating Theater: Protocol for Scoping Review
Source: JMIR Res Protoc. 2025 Feb 12;14:e67660. doi: 10.2196/67660 (PMC11888008; doi:10.2196/67660)
Supplement: Multimedia Appendix 9 [file resprot_v14i1e67660_app9.docx]

**Textbox S1:** Data extracted from studies

| **General information** | **Methods** | **Characteristics** | **Results** |
| --- | --- | --- | --- |
| Author’s name | Design | Location | Positive |
| Publication year | Participants  Characteristic | The perioperative period | Negative |
| Country | Sampling and analyse | Team Framework/model | Level of proof |
|  |  | Patient partnership |  |
|  |  | Implementation strategies |  |
|  |  | Triggers |  |
|  |  | Cognitive aid |  |
|  |  | Nursing Care concept |  |
|  |  | Outcome process |  |
|  |  | Outcome patient |  |
|  |  | Key finding |  |
